# 

**Published:** 1848-05

**Authors:** 


*Termination of the third Volume of this Journal.*—Our subscribers will observe that with this number terminates the *third* volume of our Journal. The title page and index for volume third is embraced in this number, intended to be detached, and placed at the commencement of the work, when bound. The present issue has been delayed a few days in order to announce some contemplated arrangements for the fourth volume, calculated to enhance the value and usefulness of the work. These arrangements, if perfected, will be announced in our next number. As the printing of the first number of the next volume will not be entered upon until it be concluded whether the Journal be continued without any alteration, it may not be issued quite so early in the month of June as usual. We reserve farther remarks suggested by the expiration of one volume, and the commencement of another, for our next number.

